# The Arabidopsis Root Tip (Phospho)Proteomes at Growth-Promoting versus Growth-Repressing Conditions Reveal Novel Root Growth Regulators

**DOI:** 10.3390/cells10071665

**Published:** 2021-07-02

**Authors:** Natalia Nikonorova, Evan Murphy, Cassio Flavio Fonseca de Lima, Shanshuo Zhu, Brigitte van de Cotte, Lam Dai Vu, Daria Balcerowicz, Lanxin Li, Xiangpei Kong, Gieljan De Rop, Tom Beeckman, Jiří Friml, Kris Vissenberg, Peter C. Morris, Zhaojun Ding, Ive De Smet

**Affiliations:** 1 Department of Plant Biotechnology and Bioinformatics, Ghent University, 9052 Ghent, Belgium; nikonorova.nat@gmail.com (N.N.); cassioflavio.fonsecadelima@psb.vib-ugent.be (C.F.F.d.L.); shzhu@psb.vib-ugent.be (S.Z.); brcot@psb.vib-ugent.be (B.v.d.C.); lamvu@psb.vib-ugent.be (L.D.V.); gieljancactus@hotmail.com (G.D.R.); tobee@psb.vib-ugent.be (T.B.); 2 VIB Center for Plant Systems Biology, 9052 Ghent, Belgium; 3 Division of Plant and Crop Sciences, School of Biosciences, University of Nottingham, Loughborough LE12 5RD, UK; drevanmurphy@gmail.com; 4 Integrated Molecular Plant Physiology Research, Biology Department, University of Antwerp, Groenenborgerlaan 171, 2020 Antwerpen, Belgium; daria.balcerowicz@uantwerpen.be (D.B.); kris.vissenberg@uantwerpen.be (K.V.); 5 Institute of Science and Technology (IST) Austria, 3400 Klosterneuburg, Austria; lanxin.li@ist.ac.at (L.L.); jiri.friml@ist.ac.at (J.F.); 6 The Key Laboratory of Plant Development and Environmental Adaptation Biology, Ministry of Education, School of Life Sciences, Shandong University, Qingdao 266237, China; kongxiangpei@sdu.edu.cn (X.K.); dingzhaojun@sdu.edu.cn (Z.D.); 7 Plant Biochemistry & Biotechnology Lab, Department of Agriculture, Hellenic Mediterranean University, Stavromenos, 71410 Heraklion, Crete, Greece; 8 Institute for Life and Earth Sciences, School of Energy, Geosciences, Infrastructure and Society, Heriot-Watt University, Riccarton, Edinburgh EH14 4AS, UK; p.c.morris@hw.ac.uk; 9 Centre for Plant Integrative Biology, University of Nottingham, Loughborough LE12 5RD, UK

**Keywords:** primary root, (phospho)proteomics, auxin, (receptor) kinase

## Abstract

Auxin plays a dual role in growth regulation and, depending on the tissue and concentration of the hormone, it can either promote or inhibit division and expansion processes in plants. Recent studies have revealed that, beyond transcriptional reprogramming, alternative auxin-controlled mechanisms regulate root growth. Here, we explored the impact of different concentrations of the synthetic auxin NAA that establish growth-promoting and -repressing conditions on the root tip proteome and phosphoproteome, generating a unique resource. From the phosphoproteome data, we pinpointed (novel) growth regulators, such as the RALF34-THE1 module. Our results, together with previously published studies, suggest that auxin, H^+^-ATPases, cell wall modifications and cell wall sensing receptor-like kinases are tightly embedded in a pathway regulating cell elongation. Furthermore, our study assigned a novel role to MKK2 as a regulator of primary root growth and a (potential) regulator of auxin biosynthesis and signalling, and suggests the importance of the MKK2 Thr^31^ phosphorylation site for growth regulation in the *Arabidopsis* root tip.

## 1. Introduction

One of the exceptional features that distinguishes plants from animals is the ability to continuously grow throughout their life. Constant root growth is essential for plant survival, water and nutrition uptake and adaptation to environmental stresses [1,2]. The source of this ceaseless growth is in the meristems that initiate the formation of new tissues and organs. The root meristem has a well-defined structure with stereotypical patterns of cell types along radial and longitudinal axes [3] and a stem cell niche to maintain growth [4,5,6,7,8]. Along the longitudinal axis, the primary root is divided into meristematic, elongation and differentiation zones [9]. For meristem maintenance and continuous root growth, the rate of cell differentiation should be equal to the rate of cell division [10,11]. Such balance is under tight control of multiple developmental triggers with a key role assigned to phytohormones [3,10,12].

Phytohormones are naturally occurring molecules that act at very low concentrations as signalling compounds and regulate plant growth and development. One group of hormones named auxins (from the Greek “to grow”) earned its name because of its ability to induce growth responses in plants. Establishment and maintenance of auxin gradients in plants control the two main components of growth, cell division and cell expansion [13,14,15]. Auxin plays a dual role in growth regulation, and, depending on the tissue and concentration of the hormone, it can both promote and inhibit division and expansion processes in plants [16,17,18,19]. Recent studies have shown that increased cellular auxin levels result in dramatically enhanced root cell elongation, altered expression of cell wall remodelling genes and reduced cell wall arabinogalactan complexity in *Brachypodium* [20,21]. A positive effect of auxin on growth was hypothesised by the acid growth theory [22,23,24]. This theory postulates that auxin triggers the activation of plasma membrane (PM)-localised H^+^-ATPases (proton pumps), resulting in acidification of the apoplast, activation of cell wall-loosening enzymes, and turgor pressure-mediated cell expansion. However, recent studies have shown that higher cellular auxin levels in *Brachypodium* roots were not related to proton pump activation or elevated proton excretion [21]. Auxin was nevertheless shown to be important for apoplast acidification and stimulation of cell expansion in the *Arabidopsis* root [18]. Furthermore, relatively high concentrations of exogenous auxin as well as endogenous elevation of auxin levels lead to root growth reduction [13,18]. Notably, both auxin treatment and activation of auxin biosynthesis result in transient apoplast alkalinisation [18]. However, the role of the apoplastic pH in root growth regulation remains unclear, since other cellular mechanisms downstream of auxin have been proposed, including microtubule re-arrangements [25] or vacuolar fragmentation [26,27]. In addition, the inhibitory effect of high auxin levels on primary root growth could be a result of auxin–ethylene crosstalk, as auxin stimulates ethylene biosynthesis [28,29] and ethylene leads to cell wall alkalinisation [30] and inhibition of root cell elongation [31].

Canonical auxin signalling starts with auxin binding to the receptor complex, followed by modulation of gene transcription and protein abundance [32,33,34]. However, recent studies also have shown an alternative mechanism in roots involving intra-cellular auxin perception, but not transcriptional reprogramming [35]. Although our understanding of the effects of auxin on *Arabidopsis* root growth at the protein and phosphorylation level is increasing [36,37,38,39,40,41,42], it remains incomplete. To address this gap in our knowledge, the aim of this study was to explore the impact of different concentrations of the synthetic auxin NAA—to obtain growth-promoting and growth-repressing conditions —on the root tip proteome and phosphoproteome.

## 2. Materials and Methods

### 2.1. Plant Materials and Growth Conditions

The following lines were used: *mkk1-2* (SALK_027645) and *mkk2-1* (SAIL_511_H01) [42], *the1-1* [43], *ralf34-1* and *ralf34-2* [44], *eru* [45] and *phb3* [7]. All T-DNA insertion lines used in the current study were in Col-0 background, apart from *ralf34-2* that was in L*er* background. Seeds (except for *phb3* and *eru*) were surface-sterilised and sown on half strength Murashige and Skoog (MS) medium, containing 1% agar and 0.8% sucrose, pH 5.8. Seeds were stratified at 4 °C in the dark for 2 days. Afterwards, seeds were germinated on vertically positioned Petri dishes containing half strength MS medium—with indicated naphthalene-1-acetic acid (NAA) concentrations—and grown for the indicated time after germination in a growth chamber at 21 °C under continuous light (100 µmol m^-2^ s^-1^ photosynthetically active radiation). For IAA treatment, 4- or 5-day-old Col-0 and *ralf34-1* seedlings were transferred to the surface of solid ½ MS (+ 1% sucrose) 1% agar medium with indicated concentrations of indole-3-acetic acid (IAA). For the *eru* mutant (SALK_083442C), seeds were surface-sterilised and sown on Gilroy medium [46] with different concentrations of NAA for 6 days. Gilroy medium always contained 0.8% phytagel and 1% sucrose at pH 5.7. Seeds were germinated on vertically positioned Petri dishes in a growth chamber at 22 °C under long-day photoperiod (16 h light, 8 h dark). For the *phb3* (SALK_020707) mutant [47], seeds were surface-sterilised by chlorine fumigation, stratified for two days at 4 °C on MS medium and grown in a growth chamber at 22 °C under long-day photoperiod. It should be noted that validation experiments were performed over different years and in different labs, each with (slightly) different growth conditions for optimal primary root growth. This is reflected in the diverse seedling ages and sometimes slightly different auxin concentrations.

### 2.2. Primary Root Length Analyses

Plates were scanned at 600 dpi resolution. Primary root length was measured using the NeuronJ plugin in Fiji package (https://fiji.sc; version 1.0). For the *eru* mutant, primary root images were recorded using a Nikon AZ100 multizoom macroscope and primary root length was measured using ImageJ. Data was analysed and visualised using R software environment (R Foundation for Statistical Computing, Vienna, Austria; https://www.r-project.org; version 3.3.3).

### 2.3. Scanner Growth Assay

The petri dishes containing the IAA-grown Col-0 and *ralf34-1* seedlings were fixed onto a vertically mounted flatbed scanner (Epson perfection V370) and seedlings were imaged through the layer of medium. Scans were taken automatically every hour using the AutoIt script described previously [48] at 800 or 1200 dpi. The resulting image series were analysed using StackReg stabilisation and the Manual Tracking plugin in ImageJ.

### 2.4. Data Analysis

Data shown in graphs are average values of multiple biological repeats (as indicated in the figure legends). Images were processed with Inkscape.

### 2.5. (Phospho)Proteome Analysis

For the NAA experiment, protein extraction, phosphopeptide enrichment, and liquid chromatography with tandem mass spectrometry (LC-MS/MS) analysis [49] was performed on 1 cm root tips of Col-0 seedlings. The proteome analysis of the complete root systems of 11-day-old *mkk1-2* and *mkk2-1* mutants was performed according to [50]. Data analysis was performed according to [49]. Where normalised phosphoproteome data was reported, we normalised all the phosphosites on all the proteins before statistical analysis in Perseus, performed statistical analysis (ANOVA with *p* < 0.05) and clustered the output.

### 2.6. Sequence Alignment

Protein sequences of CrRLK1 family members were aligned using a progressive alignment algorithm [51] to create multiple sequence alignments with CLC DNA Workbench 7 using the following settings: Gap open cost (10), Extension cost (1), End gap cost (as any other), Alignment (very accurate).

### 2.7. Accession Numbers

All MS proteomics data were deposited to the ProteomeXchange Consortium via the PRIDE partner repository [52] with the data set identifier PXD021267. All of the MS/MS spectra can be accessed in MS-Viewer [53] using the search key rjfbkn8oqd (auxin phosphoproteome), vddjji7zda (auxin proteome) and 7mbvmfizrz (*mkk1 mkk2* proteome).

## 3. Results and Discussion

### 3.1. Establishment of Optimum NAA Levels for Repression and Promotion of Primary Root Growth

To identify the optimum concentrations of NAA, a synthetic auxin analogue, that promotes or inhibits *Arabidopsis* primary root growth, we measured the primary root length of seedlings that were grown in the absence or presence of 0.1, 10 and 100 nM NAA for 11 days after germination (in our study, radicle protrusion took place 4 days after stratification and imbibition). This root assay revealed that 0.1 nM NAA promoted primary root growth; however, constant exposure to 10 and 100 nM NAA caused primary root growth reduction (Supplementary Figure S1A). Taken together, this root assay indicated that, at a low concentration, NAA promotes primary root growth and that, at a high concentration, NAA caused growth reduction. However, so far, it remained largely unclear what the underlying molecular mechanisms controlling these opposite responses are.

### 3.2. Proteome and Phosphoproteome Profiling to Unravel Concentration-Dependent Root Growth NAA Response

To gain insight into the molecular changes associated with growth-promoting and growth-repressing conditions associated with low and high NAA concentrations, respectively, we focused on changes in the proteome and phosphoproteome in the *Arabidopsis* root tip. *Arabidopsis* seedlings were grown vertically on solid ½ MS medium containing different concentrations of NAA (mock, 0.1, 10 and 100 nM) and five biological replicates of 1 cm root tips were harvested from 11 days after germination seedlings. We chose a 1 cm root tip, as in this zone we could capture the effect of NAA on both cell division and cell expansion, but at the same time avoid the region with NAA-induced lateral root formation (Supplementary Figure S1B). We furthermore chose 11-day-old seedlings, as we detected a difference in primary root growth upon NAA treatment at this age in agreement with a (steady state) root growth promotion or repression. From these root tips, proteins were extracted and used for two parallel analyses: (i) the total proteome, enabling us to identify key proteins responding to NAA concentration gradients, and (ii) the phosphoproteome, allowing us to gain insights into the NAA concentration-dependent phosphorylation events.

**Figure 1:**
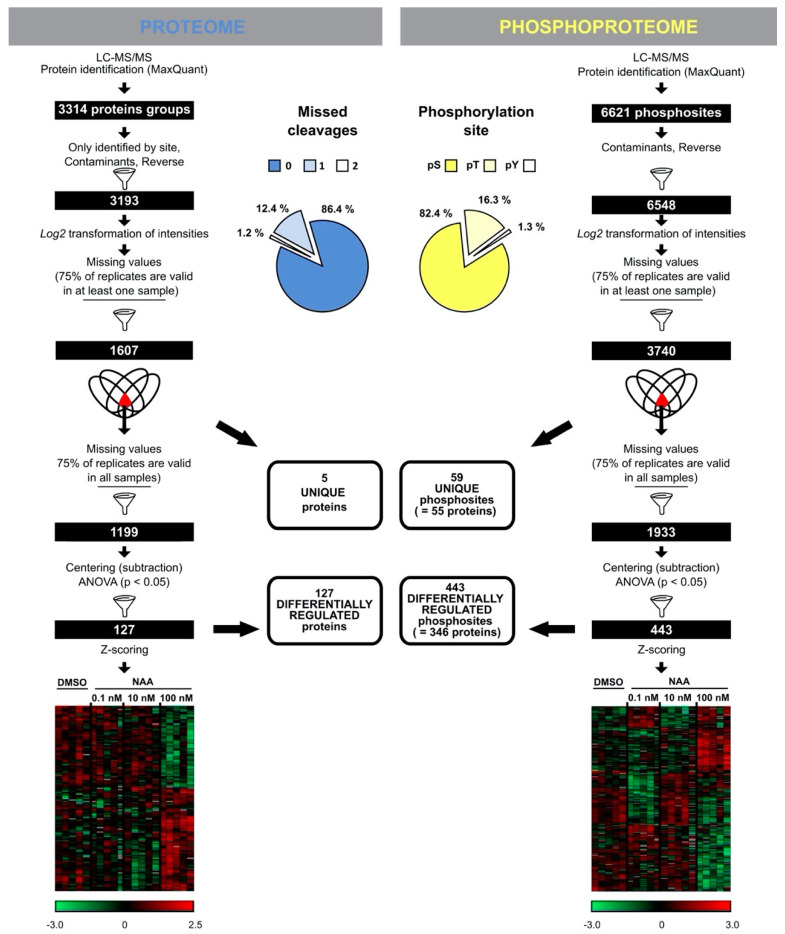
NAA-triggered protein and phosphoprotein changes. Workflow illustrating the steps to obtain a reliable set of proteins or phospho-sites following LC-MS/MS. Venn diagrams indicate steps where “unique” proteins (not at all detected in at least one of the treatment conditions)/phosphosites (detected in maximum one out of five replicates of at least one condition) (with corresponding numbers) were filtered out from the statistical analysis. Heatmaps depict statistically significant proteins and phosphosites based on Pearson correlation (see Supplementary Figures S3 and S8 for hierarchical clustering). Centred Z-scored log2-transformed intensity values on heatmaps are colour-coded according to provided colour gradient scales. The blue pie chart shows missed cleavages of tryptic peptide bonds for the proteome data. The yellow pie chart shows the percentage of identified phosphorylated serine (pS), threonine (pT) and tyrosine (pY) residues.

**Figure 2:**
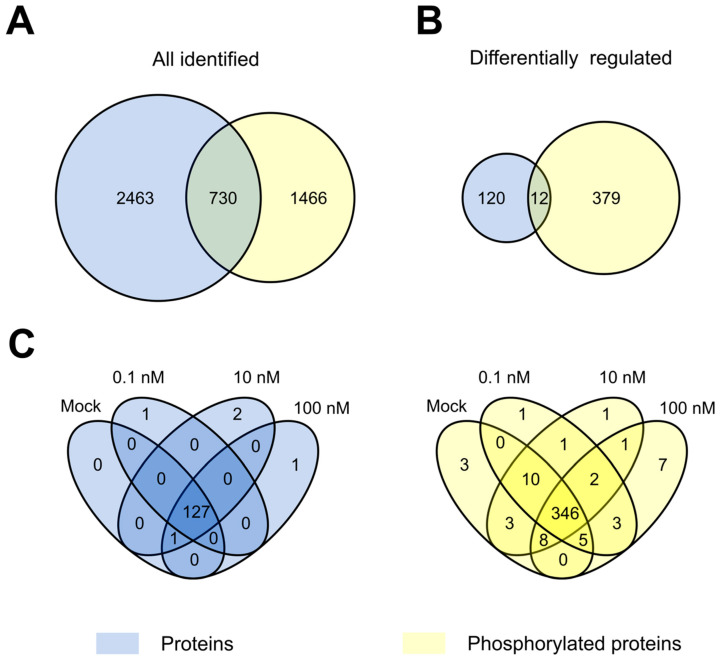
Comparison of NAA proteome and phosphoproteome data. (**A**,**B**) Overlap of all identified and differentially regulated proteins (blue; 3193 protein groups) and phosphoproteins (yellow; 6548 phosphosites mapped to 2196 proteins) in proteome and phosphoproteome datasets. (**C**) Overlap between differentially regulated (including “unique” ones) proteins (blue) or phosphoproteins (yellow) at different NAA concentrations.

The proteome analysis of control and NAA-treated samples displayed 13.6% missed cleavages and resulted in the identification of 3193 protein groups (a protein group includes proteins that cannot be unambiguously identified by unique peptides but have only shared peptides) (Figure 1 and Supplementary Table S1). The phosphoproteome analysis led to the identification of 6548 phosphorylated sites (belonging to 2196 proteins and distributed over 82.4%, 16.3% and 1.3% phosphorylated serine, threonine and tyrosine, respectively) (Figure 1 and Supplementary Table S2). Statistical analysis of the proteome data determined 127 differentially abundant proteins after NAA treatment (ANOVA *p*-value < 0.05) (Figure 1 and Supplementary Table S1). In addition, five differentially abundant proteins were not at all detected in at least one of the treatment conditions (Figure 1 and Supplementary Table S1). At the same time, analysis of the phosphoproteome dataset revealed 443 differentially abundant phosphopeptides (ANOVA *p*-value < 0.05) that could be mapped on 346 proteins and 59 phosphorylated peptides that are derived from 55 proteins (detected in maximum one out of five replicates of at least one condition) (Figure 1 and Supplementary Table S2). Interestingly, our data indicated that NAA-mediated growth responses appeared to be more pronounced at the level of phosphorylation of proteins rather than changes in protein abundance (Figure 2A–B). Specifically, the proportion of differentially regulated proteins was only 4% from the total identifications, while this was 18% for the proteins with altered phosphorylation. Furthermore, at the level of protein abundance, there were very few differential concentration-specific proteins, while this was more pronounced at the level of the phosphoproteins (Figure 2C).

### 3.3. The Arabidopsis Root Tip Proteome at Growth-Promoting and -Repressing Conditions

Using the PLAZA 4.0 platform [54], GO enrichment analysis was conducted on the 132 differentially regulated proteins in the context of a biological process (Supplementary Figure S2). The GO enrichment on biological processes showed that differentially regulated proteins were involved in processes such as regulation of cell wall organisation and biogenesis, negative regulation of growth, and response to H_2_O_2_ (with a 2-fold log2 enrichment cut-off). Hierarchical clustering of differentially regulated proteins revealed two large clusters of proteins mainly up- or downregulated in samples treated with the root growth-inhibiting concentration of NAA (100 nM) (Supplementary Figure S3). This could explain the enriched GO terms such as negative growth regulation and stress responses.

In this study, we wanted to unravel the differences in processes underlying growth promotion and inhibition upon an NAA concentration gradient. Thus, we focused on candidates that were: (i) exclusively present/absent in samples treated with 0.1 and 100 nM NAA, and (ii) gradually changing their abundance along the increase/decrease of NAA concentration, with both direct and inverse correlation of abundance to the NAA concentration gradient (Supplementary Figure S4).

A first group of proteins was potentially involved in root growth-promoting responses (Supplementary Figure S6 and Supplementary Table S3). For example, RAB GTPASE HOMOLOG G3F (RABG3F, AT3G18820) [55], which was present only at 0.1 nM NAA, and RNA-BINDING PROTEIN 47B (RBP47B, AT3G19130) [56], which was absent only at 0.1 nM NAA (Supplementary Figure S5A–B). A second group of proteins was potentially involved in growth inhibition responses (Supplementary Figure S6 and Supplementary Table S4). For example, PEROXIDASE 2 (PRX2, AT1G05250) [57,58] was exclusively present in samples treated with 100 nM NAA (Supplementary Figure S5C). Altogether, our proteome data set highlights a tight control of the balance between cell wall loosening and stiffening by NAA in a concentration-dependent manner, and suggests that the regulation of reactive oxygen species seems to play an important role during NAA-mediated primary root growth.

### 3.4. The Arabidopsis Root Tip Phosphoproteome at Growth-Promoting and -Repressing Conditions

GO enrichment analysis of biological process terms revealed that proteins with differentially regulated phosphosites under NAA treatment (including “unique” phosphosites) were involved in various processes, including regulation of cell growth (Supplementary Figure S7). Hierarchical clustering of 443 differentially abundant phosphosites revealed eight clusters (Supplementary Figure S8A). The two biggest clusters contained more than half of the differentially phosphorylated sites (238 sites = 54%), of which 103 and 135 were specifically up- and downregulated, respectively, in samples treated with the highest NAA concentration. Next, two large clusters representing a third of the differentially abundant phosphosites (135 sites = 31%) were up- or downregulated at both growth-promoting (0.1 nM) and growth-inhibiting (100 nM) NAA concentrations, and they likely include general growth regulators not directly linked to growth promotion or inhibition. In addition, two other clusters contained 57 sites that changed their phosphorylation status only after treatment with the growth-promoting NAA concentration. Taken together, a first set of differential phosphosites pinpointed proteins potentially involved in growth promotion responses (Supplementary Figure S9 and Supplementary Table S2 and S6) and a second set of differential phosphopeptides pinpointed proteins potentially involved in growth inhibition responses (Supplemental Figure S9 and Supplementary Tables S2 and S7). This included phosphopeptides belonging to auxin transport regulators, such as PIN-FORMED 2 (PIN2, AT5G57090) and ZINC INDUCED FACILITATOR-LIKE 1 (ZIFL1, AT5G13750) [59]. Additionally, we clustered differentially abundant phosphosites normalised to protein abundances (Supplementary Table S5 and Supplementary Figure S8B). The normalisation to protein abundances revealed, for example, differentially phosphorylated phosphopeptides of two 26S proteasome regulatory subunits RPN2a (AT2G32730) and RPN3a (AT1G20200). Phosphosites of both regulatory subunits were upregulated upon auxin. However, Ser^896^ from RPN2a (AT2G32730) was more phosphorylated at the growth-promoting NAA concentration and Ser^14^ from RPN3a at the growth-inhibiting NAA concentration.

### 3.5. Validation of Selected Candidates

To evaluate whether our proteome and phosphoproteome data sets can pinpoint novel regulators of primary root growth, we selected a subset of proteins that showed NAA-mediated differential abundance or phosphorylation and evaluated the primary root growth of respective mutants upon auxin treatment.

**Figure 3:**
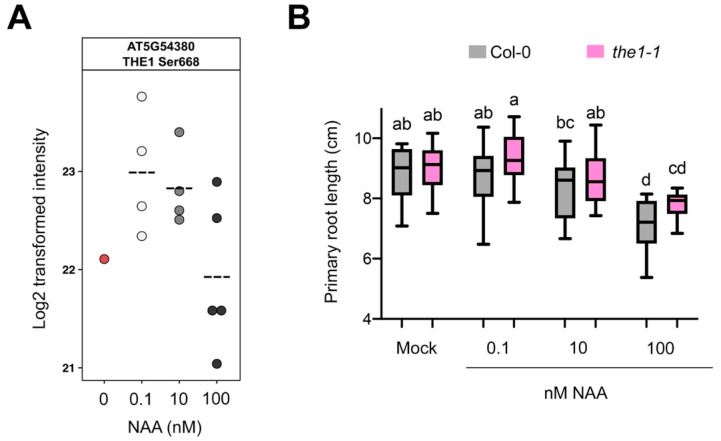
THE1 impacts NAA-controlled primary root growth. (**A**) THE1 phosphoprofile for the TGPSLDQT(0.007)HVS(0.959)T(0.034)AVK phosphopeptide upon NAA treatment. Dashed line indicates mean. Each dot is a biological replicate. (**B**) Primary root growth of 11-day-old *the1-1* seedlings in response to different concentrations (n = 18–30 seedlings). Boxplots show average with Tukey-based whiskers. Letters indicate significant difference according to two-way ANOVA with Tukey post-hoc test (*p* < 0.05).

First, we identified 17 potential protein kinases for which phosphorylation sites were significantly regulated by NAA, including four members of MITOGEN-ACTIVATED PROTEIN KINASE (MAPK) cascades and several receptor-like kinases (Supplementary Table S8). With respect to the receptor-like kinases, we first focused on THESEUS1 (THE1, AT5G54380), which plays an important role in hypocotyl elongation [43]. *THE1* is transcriptionally induced by brassinosteroids (BR), another growth-regulating plant hormone [60], and *THE1* is expressed in the elongation zone of primary roots [43]. The THE1 phosphopeptide containg Ser^668^ was detected upon NAA treatment and largely absent in mock samples (Figure 3A). The Ser^668^ is located in the protein kinase domain and highly conserved among members of the *Catharanthus roseus* RECEPTOR-LIKE KINASE 1 (*Cr*RLK1) family (Supplementary Figure S10). For our analysis, we selected the *the1-1* knockout allele containing a point mutation at a conserved residue of the extracellular domain [43]. While the primary root of 11-day-old *the1-1* seedlings was similar to Col-0 in mock conditions, it was significantly longer at growth-promoting or -repressing NAA concentrations, compared to Col-0 (Figure 3B). It should, however, be noted that the difference in length is only 7.1%, 4.3% and 9.4% for 0.1, 10 and 100 nM NAA, respectively. This could possibly be explained by functional redundancy of THE1 with related receptor-like kinases. In this context, we also identified a phosphopeptide belonging to ERULUS (ERU) (Supplementary Figure S11A), another member of the *Cr*RLK1 family [45]. However, based on the *ERU* expression pattern and previously reported phenotypes, the role of ERU is likely only associated with root hair and pollen tube tip-growth [45,61]. Nevertheless, the primary root of 6-day-old *eru* seedlings was slightly longer than Col-0 in mock conditions and at 0.1 and 10 nM NAA (Supplementary Figure S11B). It should, however, be noted that the difference in length is only 5.3%, 9.1% and 14.5% for 0, 0.1, and 10 nM NAA, respectively. It is likely that THE1 and/or ERU act redundantly with other root-expressed *CrRLK1* family members.

**Figure 4:**
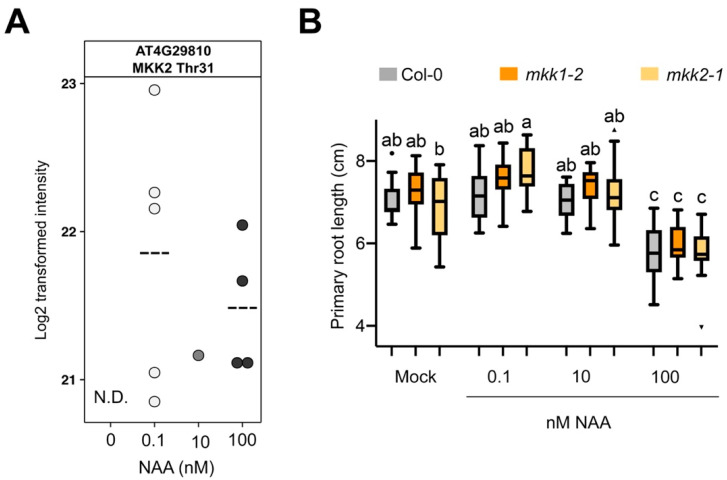
MKK2 and NAA-controlled primary root growth. (**A**) MKK2 phosphoprofile for the FLT(0.001)QS(0.031)GT(0.968)FK phosphopeptide upon NAA treatment. Dashed line indicates mean. Each dot is a biological replicate. N.D., not detected. (**B**) Primary root growth of *mkk1-2* and *mkk2-1* at different NAA concentrations (n = 10–16 seedlings) at 9 days after germination. Boxplots show average with Tukey-based whiskers and outliers (as dots or triangles). Letters indicate significant difference according to two-way ANOVA with Tukey post-hoc test (*p* < 0.05).

Eukaryotic MAPK cascades act downstream of receptors or sensors to transduce various extracellular stimuli [62,63,64]. Our list of differentially phosphorylated proteins contained several members of MAPK cascades: two MAPKKK7, MKK2 and MAPK8 (Supplementary Table S8). The *Arabidopsis* genome contains 60 MAPKKKs, 10 MAPKKs and 20 MAPKs, and some of the members of this kinase family are known to be functionally redundant [64]. Given that complexity, we focused our attention on the “bottleneck” of MAPK cascades, a member of the MAPKKs that we identified in our study, namely MKK2. Previously, MKK2 has been associated with biotic and abiotic stresses in several studies [65,66,67,68]. Our data shows that phosphorylation of MKK2 at the conserved Thr^31^ was reproducibly associated with NAA treatment 0.1 and 100 nM (Figure 4A and Supplementary Figure S12). The conserved Thr^31^ in the MKK2 protein suggests that the phosphorylation of this residue may play an important regulatory role, for example in banana during cold stress response [67]. Considering that MKK2 is closely related to and has high sequence similarity to MKK1 [42], we included mutants for both MKK1 and MKK2 in our further analysis. We evaluated primary root growth of loss-of-function mutants *mkk1-2* (SALK_027645) and *mkk2-1* (SAIL_511_H01) in response to different NAA concentrations. At 9 days after germination, *mkk2-1* demonstrated higher sensitivity to the growth-promoting NAA concentration (Figure 4B). It should be noted that the difference in primary root length for *mkk2-1* is only 8.0% for 0.1 nM NAA, compared to Col-0. However, this might be explained by functional redundancy of these two members of MAPK cascade [42]. Unfortunately, the *mkk1 mkk2* double mutant is severely impaired in its growth and development and often displays lethality at seedling stage [42,69], making it difficult to examine primary root growth responses to different NAA concentrations. Moreover, treatment with a growth-inhibiting NAA concentration (100 nM) did not reveal any differences in *mkk1*-2 or *mkk2-1* primary root growth compared to wild type (Figure 4B).

In addition, we observed an NAA concentration-dependent downregulation of PROHIBITIN3 (PHB3) protein levels (Supplementary Figure S13A). PHB3 coordinates cell division and differentiation in the root apical meristem through restricting the spatial expression of ETHYLENE RESPONSE FACTOR (ERF) transcription factors 115, 114, and 109 [7]. Indeed, a *phb3* mutant showed a short primary root in 5-day-old seedlings (Supplementary Figure S13B), indicating that the selection of candidates based on a differential protein level analysis allows novel primary root growth regulators to be identified.

### 3.6. The THE1 Ligand RALF34 Controls Auxin-Dependent Primary Root Growth

**Figure 5:**
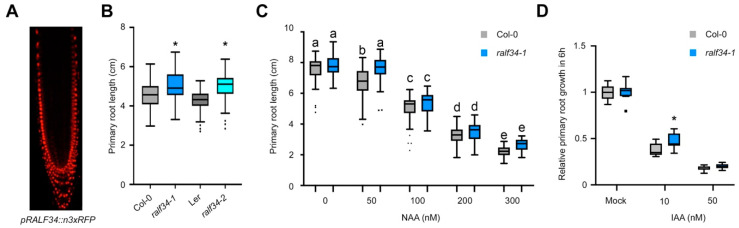
RALF34 impacts auxin-controlled primary root growth. (**A**) *RALF34* expression in the primary root tip as visualised through *pRALF34::n3xRFP* in 7-day-old seedlings. (**B**) Primary root length (cm) of 7 days after germination seedlings: wild type Col-0 (n = 133), L*er* (n=40), and RALF34 T-DNA insertion mutants *ralf34-1* (n = 104) and *ralf34-2* (n = 120). Boxplots with Tukey-based whiskers and outliers (dots) show data from three biological replicates. Asterisks indicate statistical significance (*p* < 0.001) based on Student’s t-test when T-DNA line is compared to its control. (**C**) Primary root length (cm) of Col-0 and *ralf34-1* seedlings 10 days after germination grown on various concentrations of NAA: 0 nM/EtOH mock (n = 63 and 48 for Col-0 and *ralf34-1*, respectively), 50 nM (n = 59 and 56 for Col-0 and *ralf34-1*, respectively), 100 nM (n = 61 and 41 for Col-0 and *ralf34-1*, respectively), 200 nM (n = 62 and 43 for Col-0 and *ralf34-1*, respectively) and 300 nM (n = 41 and 24 for Col-0 and *ralf34-1*, respectively). Boxplots with Tukey-based whiskers and outliers (dots) show data from 2 biological replicates. Letters indicate significant difference according to two-way ANOVA with Tukey post-hoc test (*p* < 0.05). (**D**) Normalised growth for 6 h (to mock condition) for 5-day-old Col-0 and *ralf34-1* seedlings treated with indicated IAA concentration. Boxplots with Tukey-based whiskers and outliers (7 < n < 11). Asterisks indicate statistical significance (*p* < 0.05) based on Student’s t-test when *ralf34-1* is compared to Col-0.

THE1 is a pH-dependent receptor for the RAPID ALKALINIZATION FACTOR 34 (RALF34) peptide and this signalling module has a role in fine-tuning lateral root initiation and in regulating hypocotyl elongation [70]. RALF peptides impact H^+^-ATPase (AHA) phosphorylation, which results in small changes in pH that affect root cell elongation [71,72]. Indeed, we found extensive NAA concentration-dependent regulation of AHA1 and AHA2 phosphorylation, on residues that affect pump activity [73], in our data set (Supplementary Figure S9D–J). Using a transgenic line harbouring the transcriptional reporter *pRALF34::n3xRFP* [44], we observed strong *RALF34* expression in the entire root cap and in the epidermis of the root meristematic and elongation zones of 7-day-old seedlings (Figure 5A). To explore a role for RALF34 in primary root growth, we analysed two T-DNA insertion lines in *RALF34* (*ralf34-1* and *ralf34-2*), which have been previously described [44]. Compared to their respective controls, Col-0 and L*er*, both *ralf34-1* (10.3%) and *ralf34-2* (18%) displayed a significantly longer primary root in 7-day-old seedlings (Figure 5B). To investigate the responsiveness of *ralf34-1* to auxin, we recorded the primary root length of the *ralf34-1* mutant in response to different concentrations of NAA. At 10 days after germination, *ralf34-1* seedlings grown in the presence of different NAA concentrations displayed slightly longer primary roots than wild type, but this difference is only significant for 50 nM NAA (13.2% longer) (Figure 5C), indicating a reduced sensitivity to growth-repressing NAA concentrations. In addition, we observed that primary root growth in the 5-day-old *ralf34-1* mutant after a 6-h treatment with 10 nM IAA showed mildly decreased auxin sensitivity, in comparison to Col-0 (Figure 5D). This indicates that RALF34, possibly redundantly with other RALFs, is a component downstream of auxin exerting its effect on primary root growth.

### 3.7. Proteome Profiling Identifies Molecular Changes Downstream of MKK1 and MKK2

**Figure 6:**
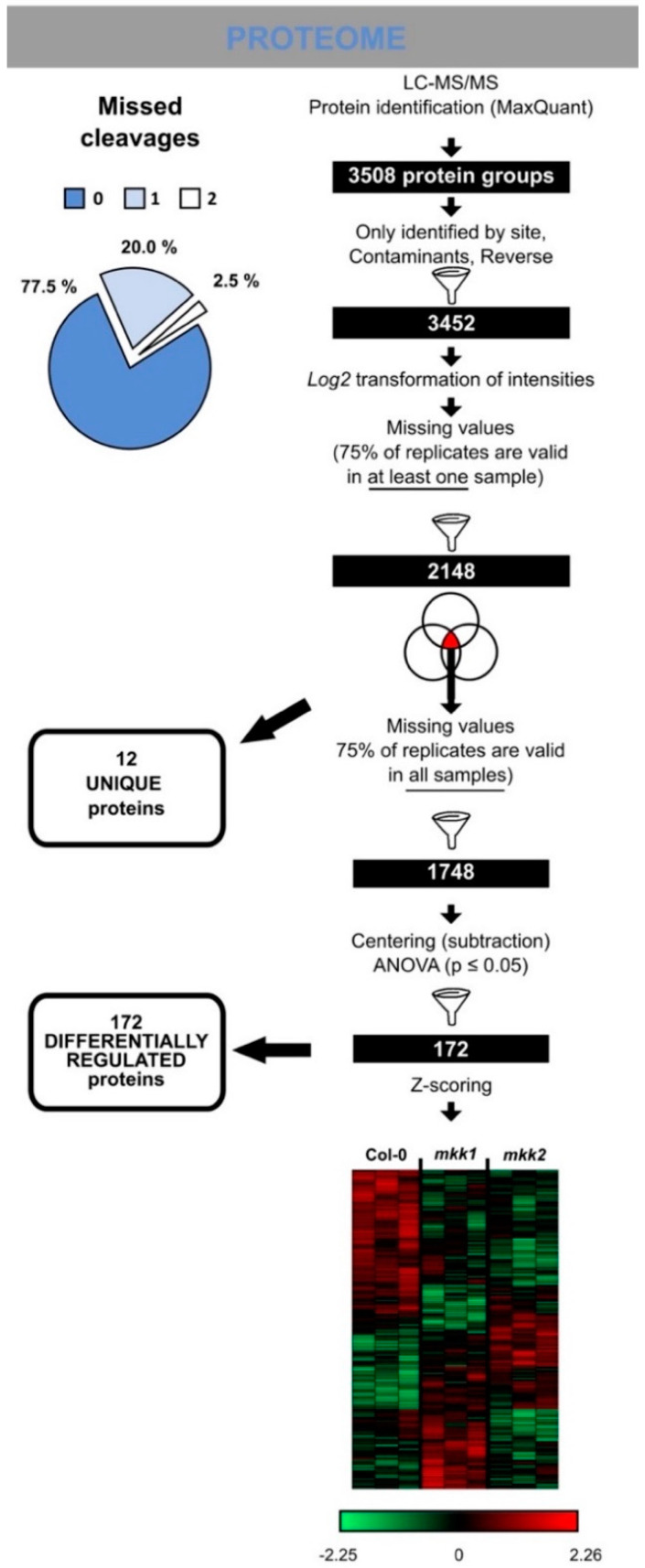
Protein changes in *mkk1-2* and *mkk2-1* mutants. Workflow illustrating the steps to obtain a reliable set of proteins or phospho-sites following LC-MS/MS. Venn diagrams indicate steps where “unique” proteins/phosphosites (with corresponding numbers) were filtered out from the statistical analysis. Heatmap depicts statistically significant proteins based on Pearson correlation. Centred Z-scored log2-transformed intensity values on heatmap are colour-coded according to provided colour gradient scale. The blue pie chart shows missed cleavages of tryptic peptide bonds for the proteome data.

**Figure 7:**
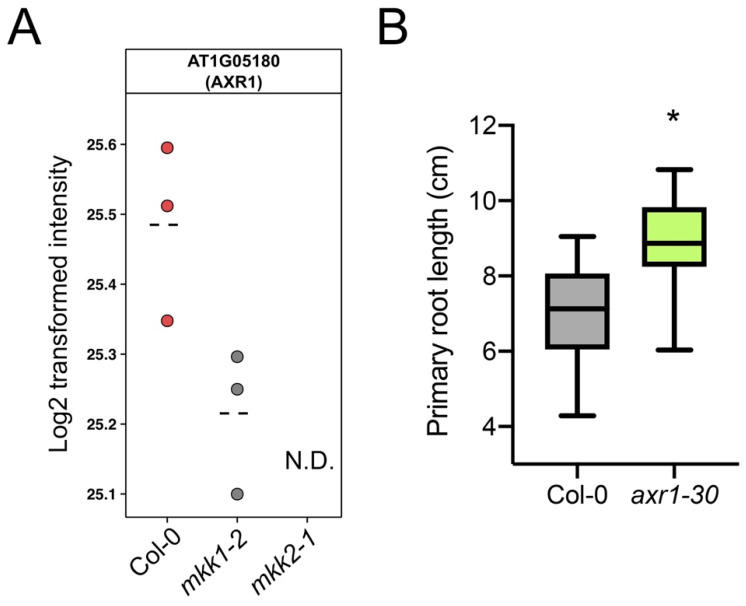
MKK1 and MKK2 affect AXR1 levels. (**A**) AXR1 protein profile in *mkk1-2* and *mkk2-1*. Dashed line indicates mean. Each dot is a biological replicate. N.D., not detected. (**B**) Primary root growth of *axr1-30* (n = 31-35 seedlings) at 11 days after germination. Boxplots show average with Tukey-based whiskers. Statistical significance (Student’s t-test) comparing *axr1-30* and Col-0 is indicated: * *p* < 0.05.

To our knowledge, MKK1 and MKK2 have not been previously implicated in primary root growth and auxin biology. Therefore, to gain insight in the molecular processes affected by a loss of function of MKK1 or MKK2, we performed proteome profiling on respective mutants in the absence of auxin. For this, total roots of 11-day-old seedlings of *mkk1-2* and *mkk2-1* mutants were harvested and proteome analysis was performed in three biological replicates. Statistical analysis revealed 172 differentially abundant proteins (Figure 6 and Supplementary Table S9). In addition, 12 proteins were not detected in at least one of the genotypes (referred to as “unique”), of which six proteins were absent in both *mkk1-2* and *mkk2-1* proteomes and two proteins, including AUXIN RESISTANT 1 (AXR1, AT1G05180), were absent in the *mkk2-1* proteome (Figure 6 and Figure 7A, Supplementary Table S9, Supplementary Figure S14).

In *Arabidopsis* leaves the MKK2-MPK10 module regulates vein complexity by altering polar auxin transport efficiency and the *mpk10* mutant has an overlapping phenotype with some mutants in auxin-related genes, for example *AUXIN-RESISTANT 1 (AXR1)* [74]. Interestingly, our data revealed that AXR1 was one of the proteins absent in the root proteome of *mkk2-1* (Figure 7A). AXR1 mediates auxin response by activating the Skp-Cullin-F-box SCF E3 ubiquitin ligase complex that targets the AUX/IAA repressors of auxin response for ubiquitination and degradation [75]. Lack of *AXR1* leads to resistance to growth-inhibiting concentrations of auxin in *Arabidopsis* roots [76] and additionally to shorter root hairs that could indicate its role in cell elongation [77]. Indeed, analysis of 11-day-old *axr1-30* mutant seedlings revealed a significant increase in primary root length (Figure 7B).

Although no large changes in phenotype and growth responses in response to NAA were observed for *mkk1-2* and *mkk2-1* (Figure 4), the root proteome data indicates altered levels of auxin signalling and biosynthesis-related proteins (Supplementary Table S9). Previously, MKK2 was found to be involved in regulation of polar auxin transport efficiency in leaves [74]. However, our data suggests that MKK2 and/or MKK1 could also be involved in signalling cascades regulating auxin biosynthesis-related proteins in the root. In the future, analysis of auxin levels in *mkk1* and *mkk2* roots will be required to support a role for MKK1 and/or MKK2 in regulating auxin biosynthesis.

## 4. Conclusions

In this study, an MS-based phosphoproteomic approach was used to identify and characterise global changes in protein abundance and phosphorylation underlying root growth promotion and inhibition by exposing seedlings to different NAA concentrations. This is a rich resource that can be used by the community to address auxin and primary root growth-related questions, and this data set is a starting point for characterising the role of specific phosphosites in those processes. In roots, canonical auxin signalling leading to modulation of gene transcription and protein abundance is debatable with respect to rapid growth responses [34,78]. Such rapid growth responses are more likely determined by plasma membrane depolarisation, Ca^2+^- and pH signalling and phosphorylation [18,22,79,80,81]. Our data also support such a mechanism of non-transcriptional auxin-mediated growth regulation. Specifically, upon auxin treatment, growth responses were much more pronounced and had more complex profiles at the level of protein phosphorylation rather than changes in protein abundance. In view of other cellular mechanisms downstream of auxin, such as microtubule re-arrangements [25] or vacuolar fragmentation [26,27], we also detected several proteins related to these processes (Supplementary Tables S1 and S2). From the phosphoproteome data, we furthermore pinpointed some (novel) growth regulators from members of receptor-like kinases and MAP kinases. Our results, together with previously published studies, suggest that auxin, H^+^-ATPases, cell wall modifications and cell wall sensing receptor-like kinases are tightly embedded in a pathway regulating cell elongation [43,45,70]. We reported a novel role for RALF34 in regulating primary root growth, but the interaction with THE1, the redundancy within CrRLK1 family members and the downstream changes remain to be investigated. Furthermore, our study assigned a novel role to MKK2 in primary root growth as a potential regulator of auxin signalling, and supports the likely importance of MKK2 Thr^31^ phosphorylation site for growth regulation in the *Arabidopsis* root tip. Further studies will shed more light on the physiological significance of these proteins and the role that the phosphorylation events play.

## Data Availability

All MS proteomics data have been deposited to the ProteomeXchange Consortium via the PRIDE partner repository [52] with the data set identifier PXD021267.

## Supplementary Materials

The following are available online at https://www.mdpi.com/article/10.3390/cells10071665/s1, Figure S1. Synthetic auxin NAA impacts primary root length; Figure S2. GO enrichment for biological processes of proteins differentially regulated upon NAA treatment; Figure S3. Main clusters of differentially regulated proteins upon NAA treatment with corresponding number of proteins; Figure S4. Schematic representation of protein and phosphopeptide behavior in the main groups of interest; Figure S5. Representative examples of “unique” proteins upon NAA treatment; Figure S6. Representative examples of proteins significantly changing abundance upon NAA treatment; Figure S7. GO enrichment for biological processes of phosphoproteins that contain differentially regulated phosphopeptides upon NAA treatment; Figure S8. Main clusters of differentially regulated phosphosites with corresponding number of phosphosites and of differentially abundant phosphosites normalised for protein abundance; Figure S9. Representative examples of “unique” phosphopeptides and AHA1/2 phosphopeptides upon NAA treatment; Figure S10. Sequence alignment of members of *Catharanthus roseus*-like receptor-like kinases 1 (CrRLK1) including THE1 and ERU; Figure S11. ERU and primary root growth; Figure S12. Sequence alignment of MKK2 homologs in across multiple plant species; Figure S13. PHB3 and primary root growth; Figure S14. Representative examples of proteins exclusively present or absent in *mkk1-2* and/or *mkk2-1* mutants; Table S1. Differentially abundant proteins upon NAA treatment; Table S2. Differentially abundant phosphosites upon NAA treatment; Table S3. GO categorisation for proteome data (potential growth promotion); Table S4. GO categorisation for proteome data (potential growth repression); Table S5. Normalised phosphoproteome; Table S6. GO categorisation for phosphoproteome data (potential growth promotion); Table S7. GO categorisation for phosphoproteome data (potential growth repression); Table S8. List of putative kinases in phosphoproteome data set; Table S9. Proteome analysis of Col-0 versus *mkk1* and *mkk2.*
