# Supplementary material for: The Arabidopsis Root Tip (Phospho)Proteomes at Growth-Promoting versus Growth-Repressing Conditions Reveal Novel Root Growth Regulators
Source: Cells. 2021 Jul 2;10(7):1665. doi: 10.3390/cells10071665 (PMC8303113; doi:10.3390/cells10071665)
Supplement: Supplementary file 1 [file cells-10-01665-s001.zip › Nikonorova_SupplementaryInformation.pdf]

SUPPLEMENTARY INFORMATION

SUPPLEMENTARY TABLES

Supplementary Table S1. Differentially abundant proteins upon NAA treatment.

Supplementary Table S2. Differentially abundant phosphosites upon NAA treatment.

Supplementary Table S3. GO categorization for proteome data (potential growth promotion).

Supplementary Table S4. GO categorization for proteome data (potential growth repression).

Supplementary Table S5. Normalized phosphoproteome.

Supplementary Table S6. GO categorization for phosphoproteome data (potential growth promotion).

Supplementary Table S7. GO categorization for phosphoproteome data (potential growth repression).

Supplementary Table S8. List of putative kinases in phosphoproteome data set.

Supplementary Table S9. Proteome analysis of Col-0 versus *mkk1* and *mkk2*.

SUPPLEMENTARY FIGURES

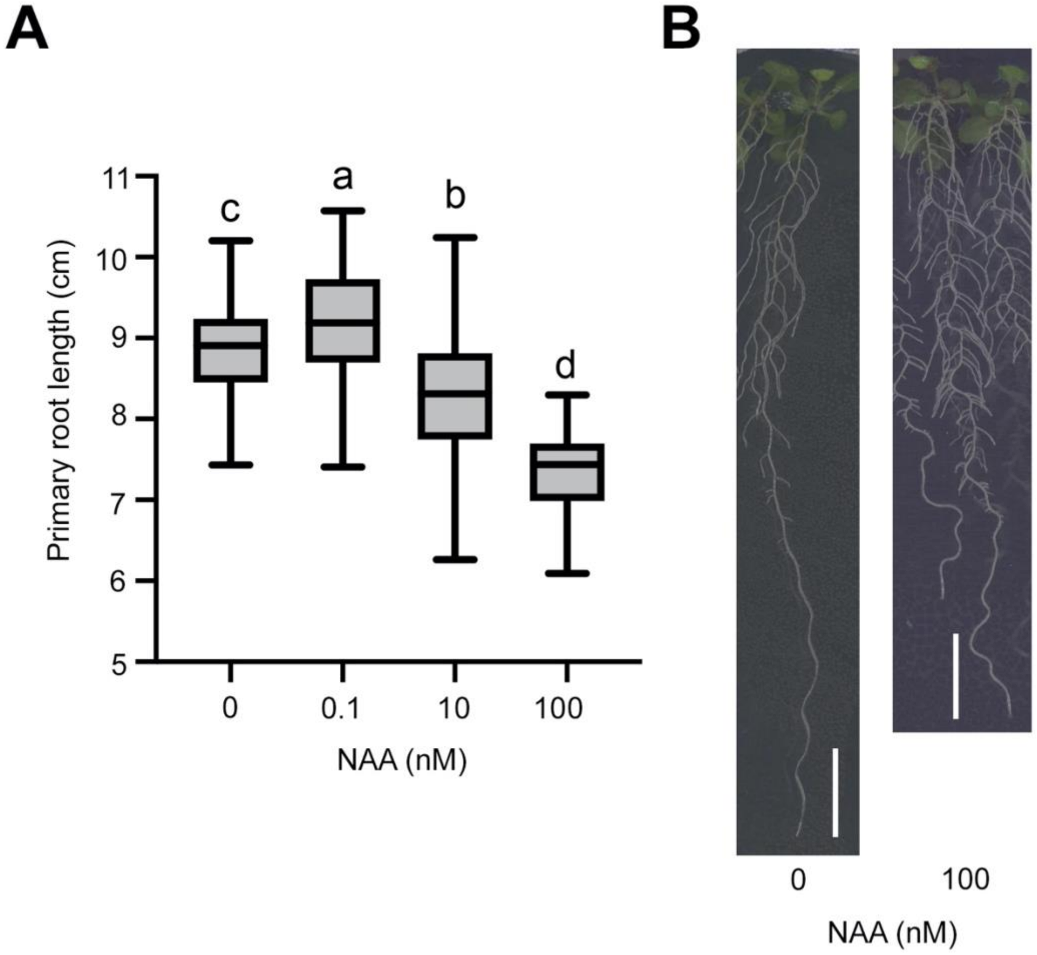

Figure S1. Auxin impacts primary root length. **(A)** Primary root length of 11-day-old *Arabidopsis* seedlings upon auxin (NAA) treatment (n = 97-135 seedlings per condition). Boxplots show average with Tukey-based whiskers. Letters indicate significant difference according to one-way ANOVA analysis ($p < 0.05$). **(B)** Representative pictures of 11-day old *Arabidopsis* seedlings at indicated auxin concentration. White bar indicates an approximate 1 cm portion that was used for (phospho)proteomics.

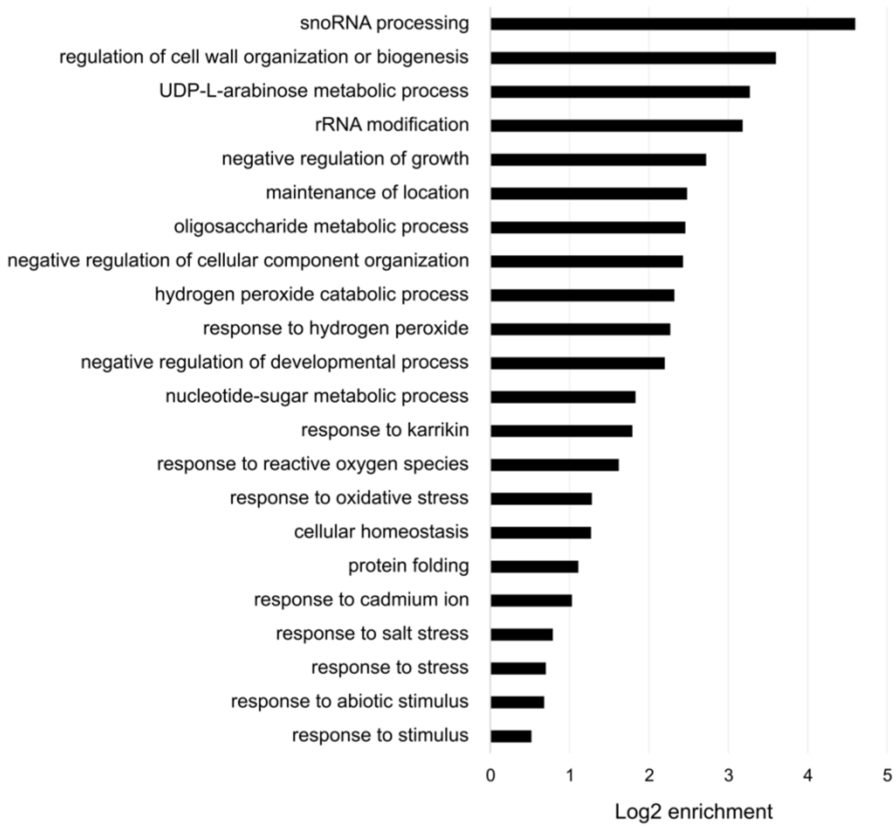

Figure S2. GO enrichment for biological processes of proteins differentially regulated upon auxin treatment.

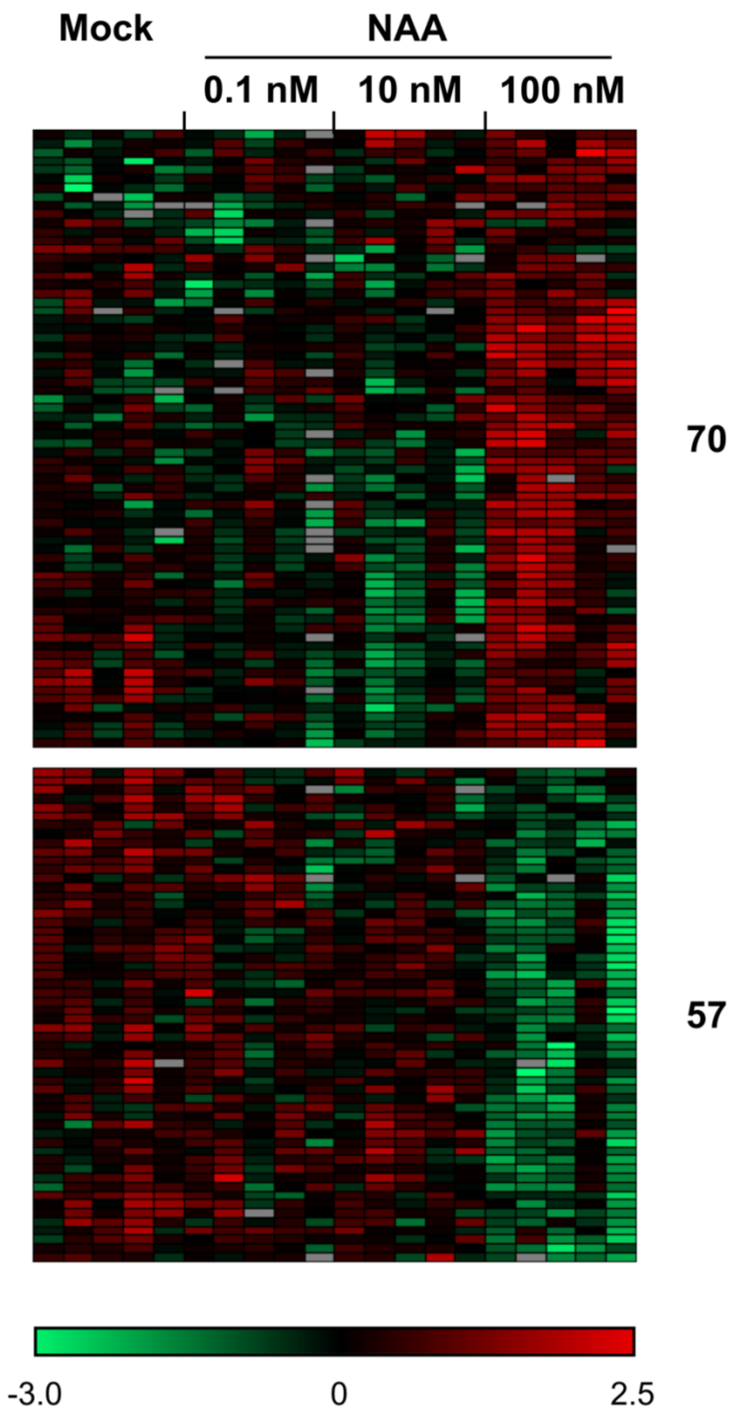

Figure S3. Main clusters of differentially regulated proteins upon auxin treatment with corresponding number of proteins. Heatmap represents a hierarchical clustering of statistically significant proteins based on Pearson correlation. Centered Z-scored log2-transformed intensity values on heatmaps are color-coded according to provided color gradient scales.

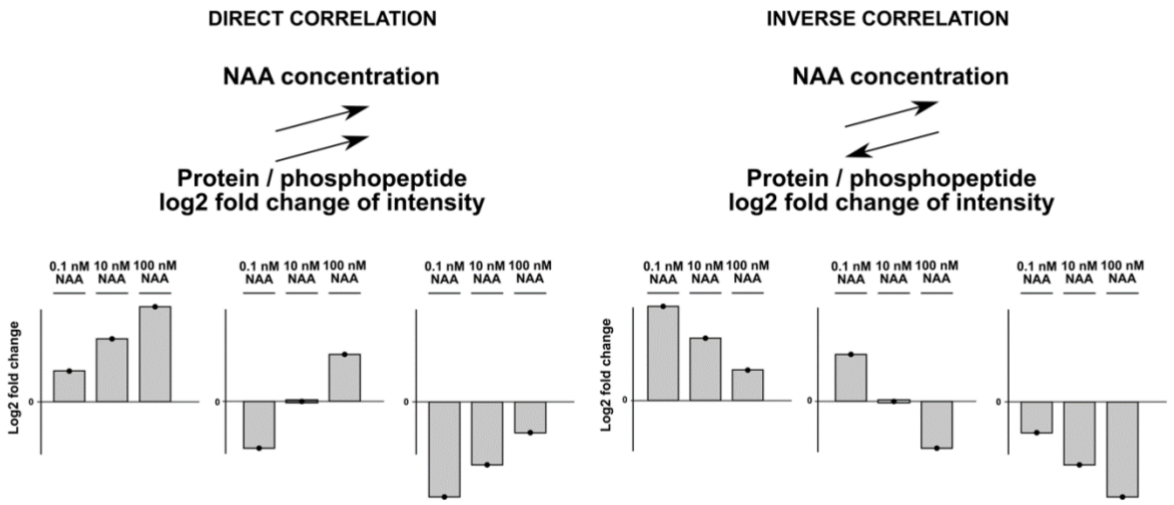

Figure S4. Schematic representation of protein and phosphopeptide behavior in the main groups of interest.

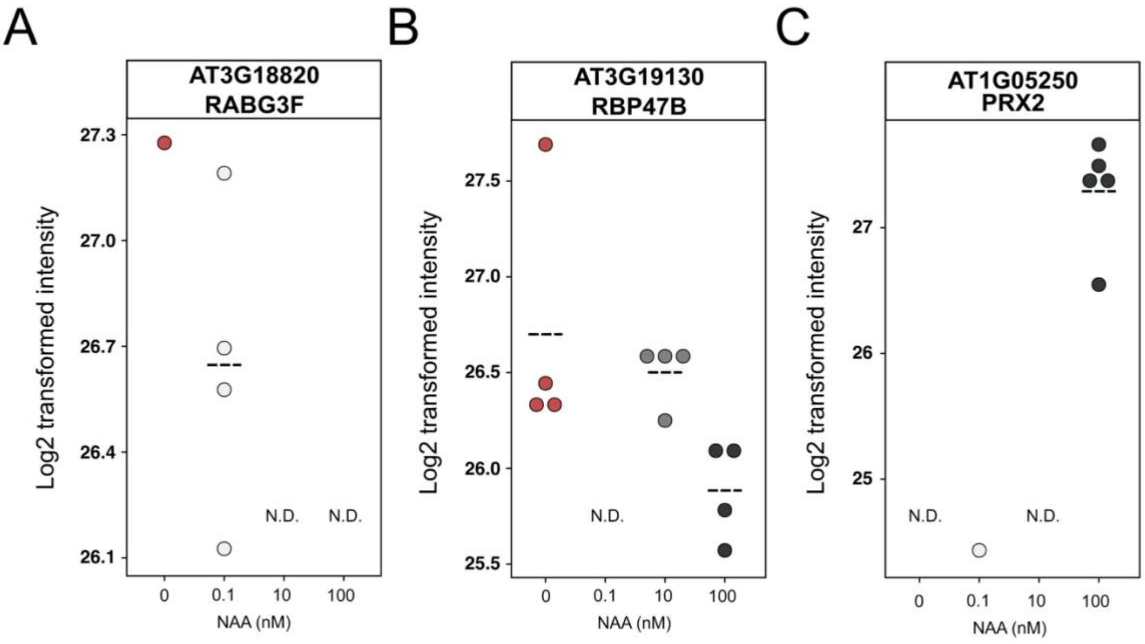

Figure S5. Representative examples of 'unique' proteins upon NAA treatment (A-C). Individual values (dots) are shown. Dashed line represents mean. N.D., not detected. In the workflow, only one detection is also considered as not reproducibly detected.

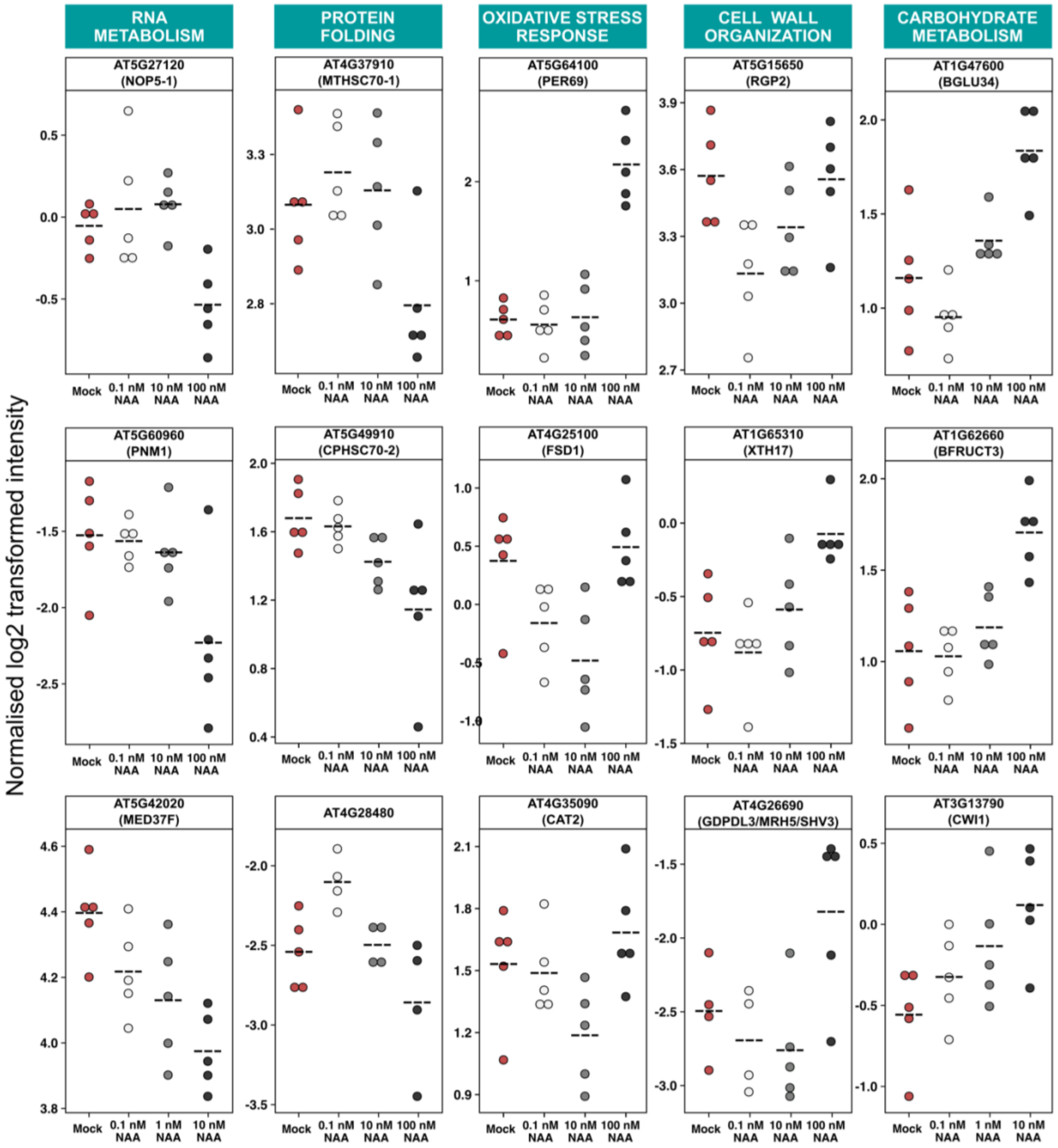

Figure S6. Representative examples of proteins significantly changing abundance upon auxin treatment. Individual values (dots) are shown. Dashed line represents the mean value.

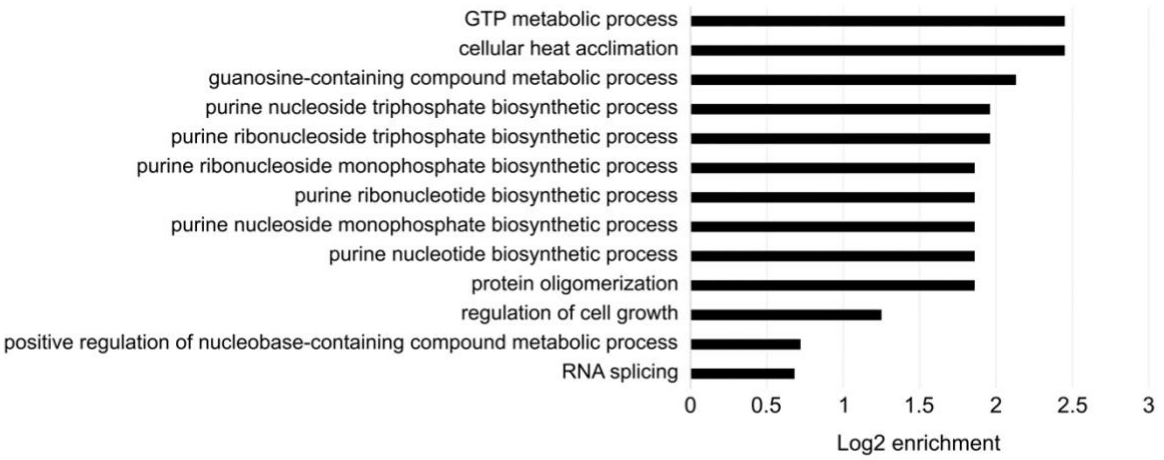

Figure S7. GO enrichment for biological processes of phosphoproteins that contain differentially regulated phosphopeptides upon auxin treatment.

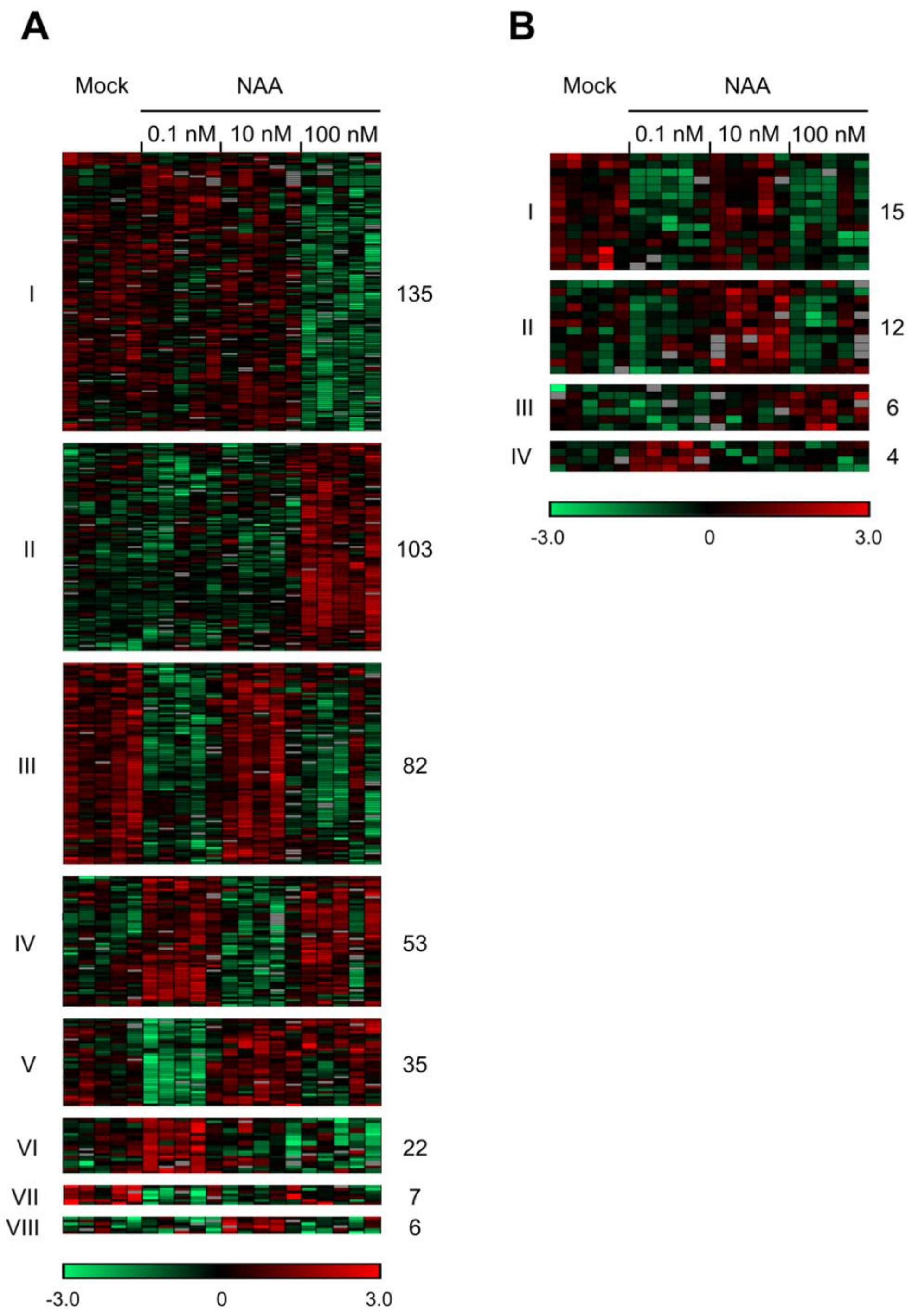

Figure S8. Main clusters of differentially regulated phosphosites with corresponding number of phosphosites (A) and of differentially abundant phosphosites normalized for protein abundance (B). Heatmap represents a hierarchical clustering of statistically significant phosphosites based on Pearson correlation. Centered Z-scored log2-transformed intensity values on heatmaps are color-coded according to provided color gradient scales.

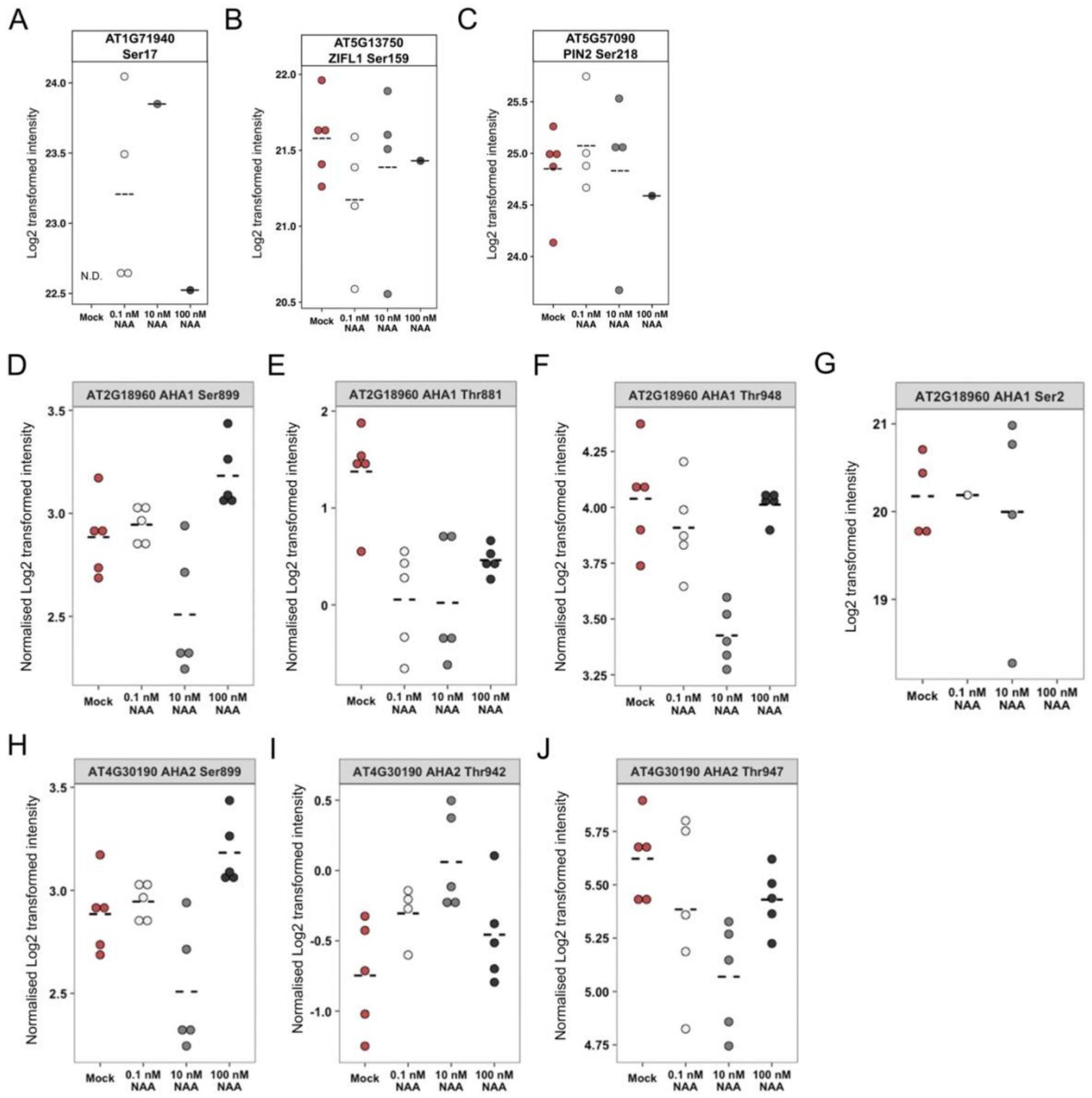

Figure S9. Representative examples of ‘unique’ phosphopeptides (A-C) and AHA1/2 phosphopeptides (D-J) upon auxin treatment. Data are for the following phosphopeptides: ILTRDEELGVIS(1)DDDDS(0.8)PS(0.2)GK (A), FNDDES(1)YDALK (B), SSAASSMIS(0.005)S(0.995)FNK (C), GS(1)YRELSEIAEQAK (D), EAQWAQAQRT(1)LHGLQPK (E), GLDIDTAGHHYT(1)V (F), S(0.995)GLEDIKNET(0.005)VDLEK (G), GS(1)YRELSEIAEQAK (H), GLDIET(0.999)PS(0.001)HYTV (I), GLDIETPSHYT(1)V (J). Individual values (dots) are shown. Dashed line represents mean. In the workflow, only one detection is also considered as not reproducibly detected.

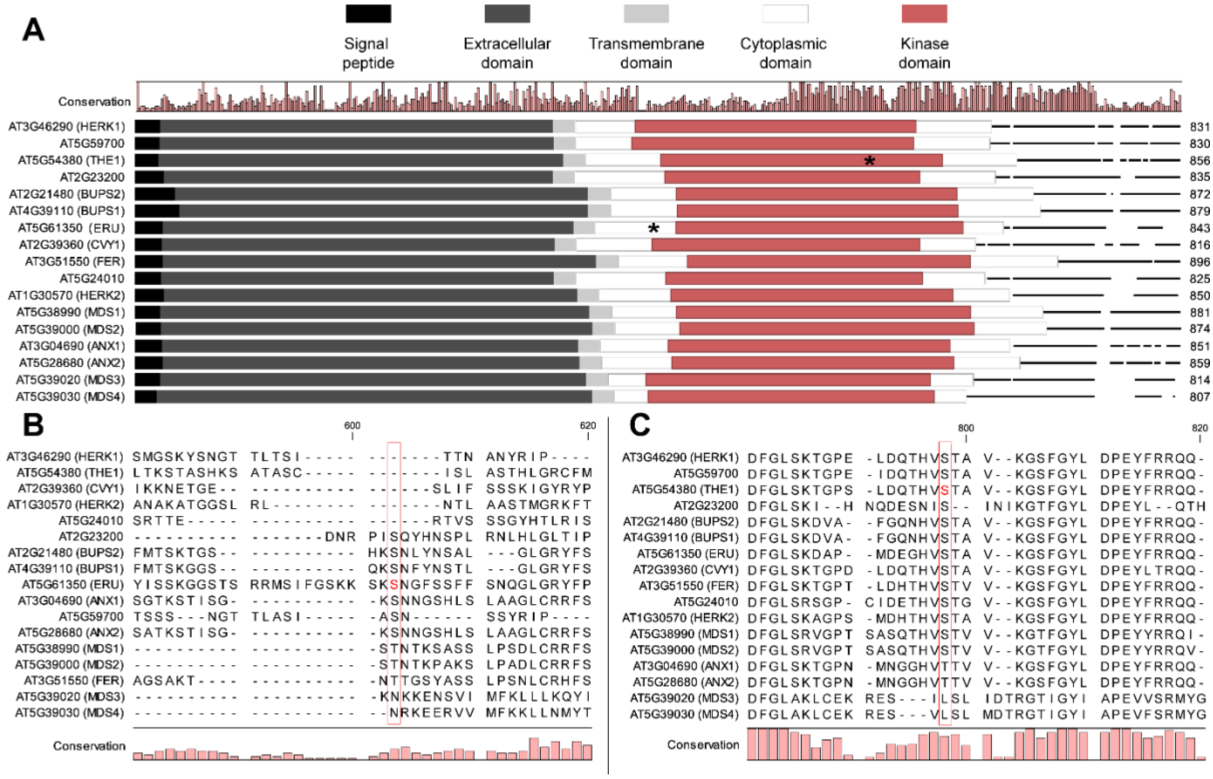

Figure S10. Sequence alignment of members of *Catharanthus roseus*-like receptor like kinases 1 (CrRLK1) including THE1 and ERU (according to Zulawski *et al.*, 2014). **(A)** Main domains of CrRLK1 members with asterisks indicating positions of phosphosites identified in the current study for THE1 and ERU. **(B-C)** Zooming in on sequence alignment with position of phosphorylated serine highlighted in red.

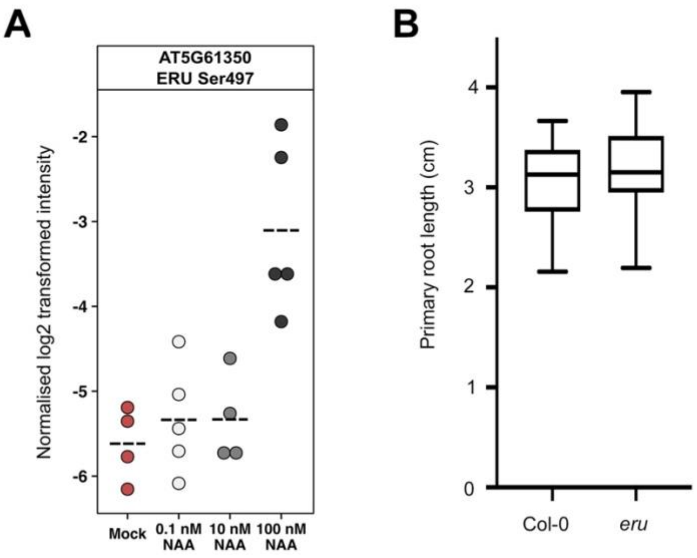

Figure S11. ERU and primary root growth. **(A)** ERU phosphoprofile for the S(1)NGFSSFFSNQGLGR phosphopeptide upon NAA treatment. Dashed line indicates mean. Each dot is a biological replicate. **(B)** The primary root length of 6-day old *eru* seedlings (n - = 30) compared to Col-0 (n = 32). Boxplots show average with Tukey-based whiskers. No significant differences were detected using an unpaired Student's t-test ($p = 0.11$).

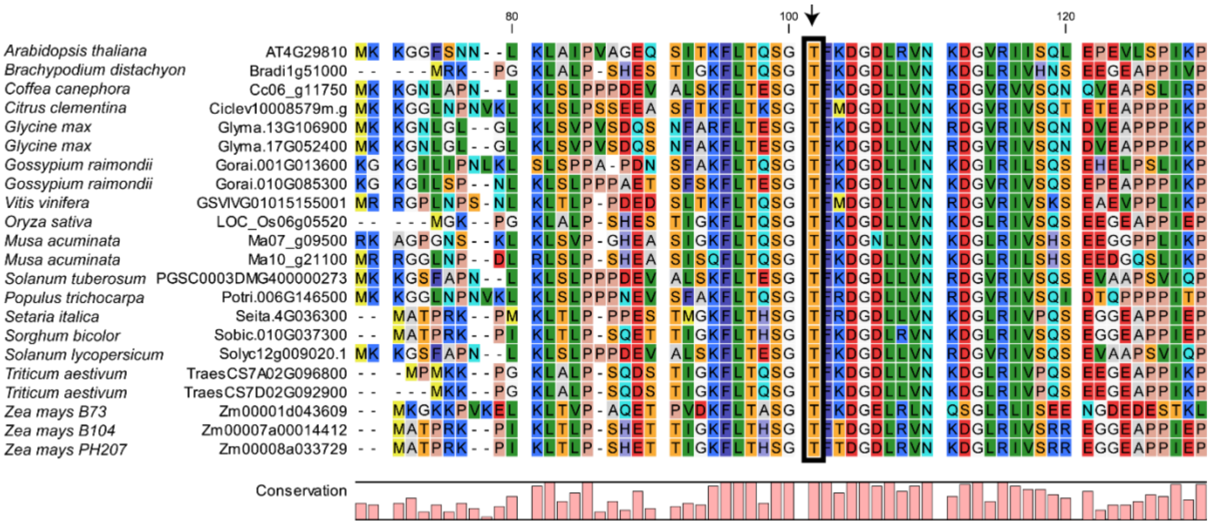

Figure S12. Sequence alignment of MKK2 homologs in across multiple plant species. Position of Thr[31] phosphorylation site is indicated by the black rectangle/arrow.

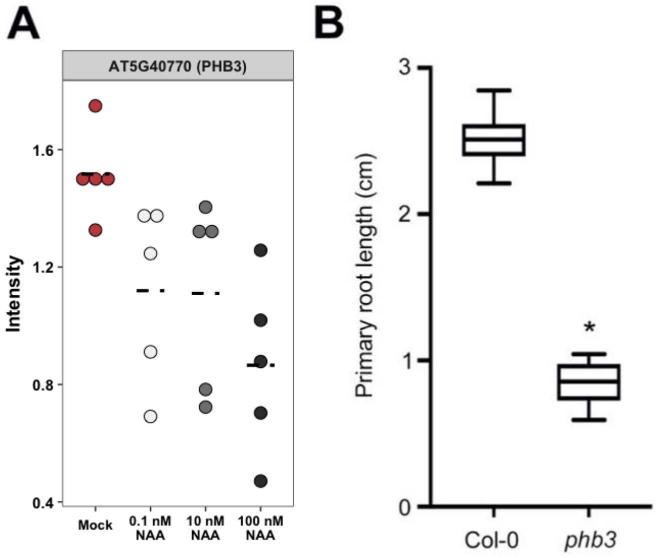

Figure S13. PHB3 and primary root growth. **(A)** PHB3 protein profile in the presence of auxin. Dashed line indicates mean. Each dot is a biological replicate. **(B)** The primary root length of 5-day-old *phb3* seedlings (n = 24) compared to Col-0 (n = 35). Boxplots show average with Tukey-based whiskers. A Student's t-test revealed a significant difference (p < 0.05).

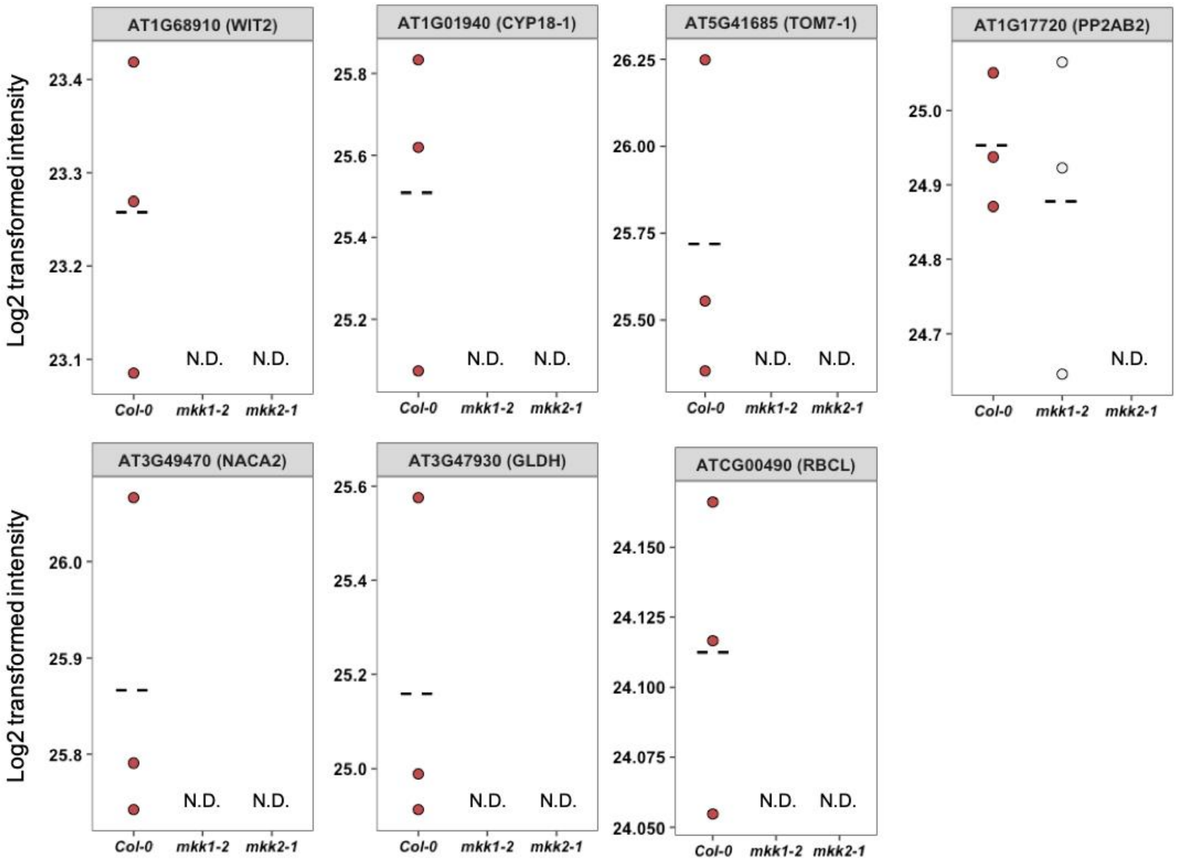

Figure S14. Representative examples of proteins exclusively present or absent in *mkk1-2* and/or *mkk2-1* mutants. Individual values (dots) are shown. Dashed line represents the mean value. N.D., not detected.
